# Optimal Phased-Array Signal Combination For Polyunsaturated Fatty Acids Measurement In Breast Cancer Using Multiple Quantum Coherence MR Spectroscopy At 3T

**DOI:** 10.1038/s41598-019-45710-1

**Published:** 2019-06-25

**Authors:** Vasiliki Mallikourti, Sai Man Cheung, Tanja Gagliardi, Yazan Masannat, Steven D. Heys, Jiabao He

**Affiliations:** 1 Institute of Medical Sciences, School of Medicine, University of Aberdeen, Aberdeen, UK; 2 Department of Clinical Radiology, Aberdeen Royal Infirmary, Aberdeen, UK; 3 Breast Unit, Aberdeen Royal Infirmary, Aberdeen, UK; 4 Department of Radiology, Royal Marsden Hospital, London, UK

**Keywords:** Breast cancer, Medical research

## Abstract

Polyunsaturated fatty acid (PUFA), a key marker in breast cancer, is non-invasively quantifiable using multiple quantum coherence (MQC) magnetic resonance spectroscopy (MRS) at the expense of losing half of the signal. Signal combination for phased array coils provides potential pathways to enhance the signal to noise ratio (SNR), with current algorithms developed for conventional brain MRS. Since PUFA spectra and the biochemical environment in the breast deviate significantly from those in the brain, we set out to identify the optimal algorithm for PUFA in breast cancer. Combination algorithms were compared using PUFA spectra from 17 human breast tumour specimens, 15 healthy female volunteers, and 5 patients with breast cancer on a clinical 3 T MRI scanner. Adaptively Optimised Combination (AOC) yielded the maximum SNR improvement in specimens (median, 39.5%; interquartile range: 35.5–53.2%, p < 0.05), volunteers (82.4 ± 37.4%, p < 0.001), and patients (median, 61%; range: 34–105%, p < 0.05), while independent from voxel volume (rho = 0.125, p = 0.632), PUFA content (rho = 0.256, p = 0.320) or water/fat ratio (rho = 0.353, p = 0.165). Using AOC, acquisition in patients is 1.5 times faster compared to non-noise decorrelated algorithms. Therefore, AOC is the most suitable current algorithm to improve SNR or accelerate the acquisition of PUFA MRS from breast in a clinical setting.

## Introduction

Breast cancer is the most prevalent form of cancer among women worldwide^1^, and polyunsaturated fatty acid (PUFA) is a key marker for treatment monitoring^2^. Although magnetic resonance spectroscopy (MRS) is a non-invasive approach for metabolite profiling^3^, PUFA measurement is not possible due to the overwhelming signals of monounsaturated fatty acid (MUFA) and water^4^. Multiple quantum coherence (MQC) MRS suppresses MUFA and water for accurate PUFA detection, but sacrifices half of the acquired signal^4,5^. Although PUFA is abundant in healthy female breast, its depletion is characteristic of rapid cell division in tumour^6^. Due to the low abundance of PUFA in tumour^6^ and further loss of half of the detected signal using MQC^4^, improvement in SNR is essential for accurate quantification in treatment monitoring, especially if small changes in PUFA concentration occur. In addition, the achieved SNR improvement can be utilised to reduce acquisition time. Therefore, it is critical to enhance the signal to noise ratio (SNR) of PUFA acquired using MQC MRS in patients with breast cancer.

The combination of signals acquired from phased array coils provides an effective approach for improving SNR^7,8^ with prior development focusing on conventional MRS in the brain (Table 1)^9–20^. Equal Weighting algorithm derives the combined spectrum through summation of signals from all the coil elements after all signals are aligned in phase^9^. To account for the influence of coil sensitivities, the Signal Weighting algorithm weights each coil element with the amplitude of the reference peak received on the corresponding coil element after phase alignment^12–14^. In order to further account for the noise variations between different coil elements, the S/N Weighting algorithm weights each coil element with the SNR of the reference peak^15–17^. Subsequently, the S/N^2^ Weighting algorithm was introduced through weighting each coil element with the ratio of signal to noise square of the reference peak, and is the optimal algorithm for combination of signals containing purely uncorrelated noise^9^.

**Table 1:** Description of the existing signal combination algorithms.

Algorithms	Description
Equal Weighting	Adding after aligning in phase
Signal Weighting	Weighting based on Signal of reference peak
S/N Weighting	Weighting based on Signal/Noise of reference peak
S/N^2^ Weighting	Weighting based on Signal/Noise^2^ of reference peak
nd-comb	1. Noise decorrelation by PCA, 2. Weighting the noise decorrelated data based on Signal/Noise of reference peak
WSVD	1. Noise decorrelation by PCA, 2. Weighting the noise decorrelated spectrum based on the first left singular vector obtained using the singular value decomposition of the noise decorrelated spectra
AOC	Weighting based on Signal of reference peak (typically water) multiplied by the inverted noise correlation matrix

Table describes the calculation of the weighting coefficient for the linear combination algorithms of Equal Weighting, Signal Weighting, S/N Weighting, and S/N^2^ Weighting and the noise decorrelated algorithms of nd-comb, WSVD, and AOC. AOC = adaptively optimised combination. Nd-comb = noise decorrelation combination, PCA = Principal Component Analysis, WSVD = whitened singular value decomposition.

Since correlated noise presents during *in vivo* data acquisition^10^, algorithms employing noise decorrelation were developed^10,18,21^. Noise decorrelation combination (nd-comb) initially employs the Principal Component Analysis (PCA) to remove correlated noise before applying the S/N Weighting algorithm^18,19^. In contrast, Whitened Singular Value Decomposition (WSVD)^21,22^ applies the singular value decomposition^23,24^ on the noise-decorrelated data to derive the weighting. Hence, WSVD exploits the whole noise-decorrelated spectra to estimate the weighting compared to the single reference peak used in nd-comb. Instead of eliminating the correlated noise as a preparation step, Adaptively Optimised Combination (AOC) algorithm derives the weighting from the amplitude of a reference peak (typically water) incorporating the inverted noise correlation matrix^10^.

Investigations on phased array combination algorithms have focused so far on conventional MRS in the brain^9–20^, with significant different spectral appearance and metabolite composition compared to breast. Furthermore, existing investigations were limited to phantoms and healthy volunteers, and hence confined to a narrow physiological variability raising the issue of their applicability in clinical setting. We therefore systematically examined current signal combination algorithms on PUFA spectra acquired using MQC MRS in breast tumours.

## Results

### Comparison of signal combination algorithms

**Figure 1:**
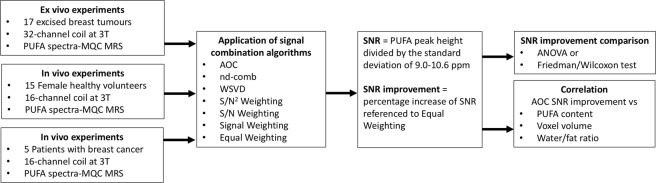
Study design. The algorithms of AOC, nd-comb, WSVD, S/N^2^ Weighting, S/N Weighting, and Signal Weighting were evaluated through PUFA spectra acquired from *ex vivo* and *in vivo* experiments. The Equal Weighting algorithm was used as a reference to calculate the SNR improvement for different algorithms. AOC = adaptively optimised combination. Nd-comb = noise decorrelation combination, WSVD = whitened singular value decomposition.

**Figure 2:**
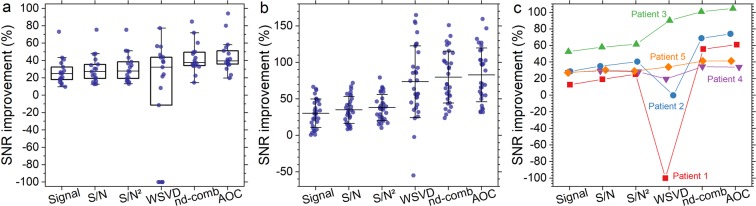
Comparison of the combination algorithms on experimental PUFA spectra. SNR improvement for (**a**) excised breast tumour specimens, (**b**) healthy volunteers, and (**c**) patients. The box plot shows the median and interquartile range while the error bar shows the mean and standard deviation of SNR improvement from each algorithm. For WSVD, the improvement was negative for 5 data out of 17 for excised breast tumour specimens, 2 data out of 30 for healthy volunteers and 2 data out of 5 for patients. Each dot represents an individual measurement. For subfigures a and b, overlapping data points are displaced horizontally to aid visual interpretation. AOC = adaptively optimised combination. Nd-comb = noise decorrelation combination, WSVD = whitened singular value decomposition.

**Figure 3:**
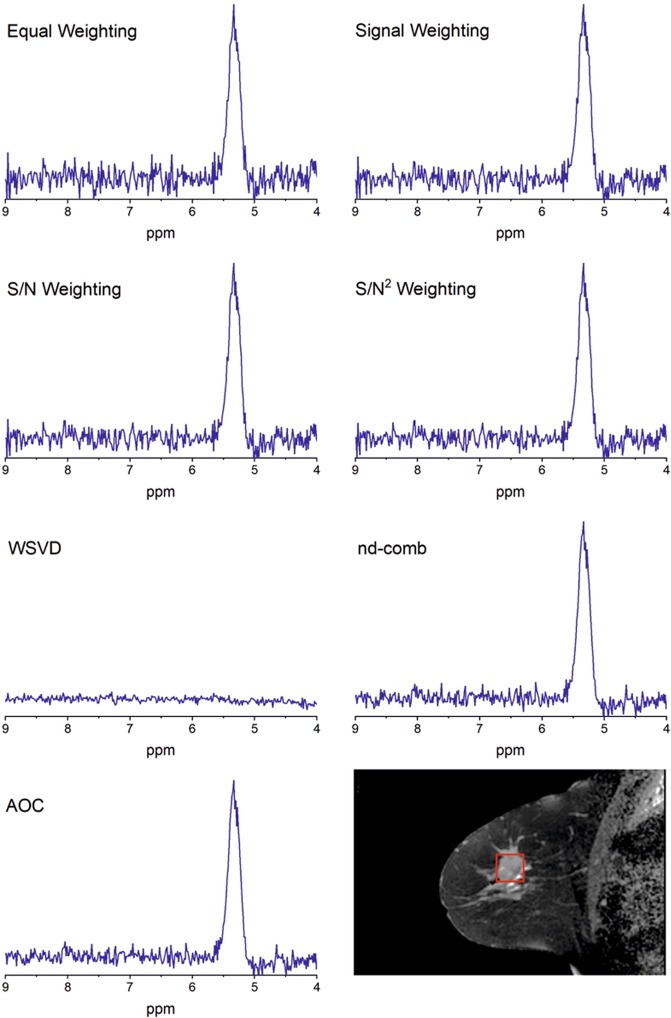
Spectra from a patient. (**a**) Combined spectra obtained from the breast tumour of a patient using AOC, nd-comb, WSVD, S/N^2^ Weighting, S/N Weighting, Signal Weighting, and Equal Weighting with SNR improvement of 60.9%, 55.8%, −100%, 25.4%, 19.2%, 12.6% respectively. (**b**) Voxel location (2 × 2 × 2 cm^3^) within an invasive ductal carcinoma, grade III, acquired from a 68-year old patient. AOC = adaptively optimised combination. Nd-comb = noise decorrelation combination, WSVD = whitened singular value decomposition, ppm = parts per million.

Signal combination algorithms^9,10,18,21^ (Table 1) were implemented and evaluated with the study design shown in Fig. 1. PUFA spectra were acquired using MQC MRS from whole breast tumour specimens freshly excised from patients, healthy female volunteers and patients with breast cancer. The combination algorithms were compared using the SNR improvement of PUFA referenced to the SNR obtained from the Equal Weighting algorithm (defined as baseline SNR). Our results show that for breast tumour specimens, the SNR improvement from AOC (median: 39.5%, interquartile range: 35.5–53.2%) was significantly higher than other algorithms (p < 0.05) (Table 2, Fig. 2a). WSVD gave negative SNR improvement for 5 out of 17 breast tumour specimens, when baseline SNR was below 7.5 showing dependency on baseline SNR and voxel volume (Supplementary Fig. S1). For spectra with baseline SNR higher than 7.5, AOC gave comparable SNR improvement to WSVD (Table 2). In healthy volunteers, SNR improvement from AOC (82.4 ± 37.4%) was significantly higher than other algorithms (p < 0.001) (Table 2, Fig. 2b). In patients, AOC yielded the highest average SNR improvement of 61% (34–105%) (median, range) (Table 2, Fig. 2c), which was significantly higher compared to each of the algorithms, apart from nd-comb (Wilcoxon signed-rank test on small sample size of N = 5). The AOC improvement was 24% (4–28%), 29% (4–35%), and 34% (5–43%) relative to S/N^2^ Weighting, S/N Weighting and Signal Weighting respectively. WSVD produced negative SNR improvement in 2 datasets acquired from healthy volunteers and 2 datasets from patients where contamination lipid signal was present, as shown in Fig. 3. AOC gave comparable SNR improvement to WSVD for healthy volunteers when spectra with negative SNR improvement were excluded (Table 2).

**Table 2:** SNR and SNR improvement for the *ex vivo* and *in vivo* experiments.

Methods	*Ex vivo* experiments			*In vivo* experiments					p-value
	Breast tumour specimens (N = 17)			Healthy volunteers (N = 15, 30 voxels)			Patients (N = 5)		
	SNR	SNR improvement %	p-value	SNR	SNR improvement %	p-value	SNR	SNR improvement %	
Equal	12.5 (6.9–18.4)	—	—	37.0 ± 21.6	—	—	9 (4–21)	—	—
Signal	14.2 (8.3–24.7)	24.8 (16.6–34.8)	<0.001	49.1 ± 33.1	29.5 ± 19.5	<0.001	12 (5–24)	28 (13–53)	0.043
S/N	14.4 (8.3–25.6)	27.2 (17.7–38.3)	<0.001	50.6 ± 33.3	34.2 ± 18.4	<0.001	12 (5–25)	30 (19–58)	0.043
S/N^2^	14.4 (8.3–26.0)	27.8 (17.9–40.8)	0.001	51.5 ± 33.3	37.4 ± 17.8	<0.001	12 (6–27)	29 (25–62)	0.043
WSVD	23.0 (13.1–32.2)	41.4 (26.1–53.6)	0.074	66.5 ± 39.6	81.3 ± 42.3	0.312	11 (7–22)	20 (−100–90)	0.043
nd-comb	16.7 (9.5–27.1)	37.7 (33.8–49.8)	0.039	64.9 ± 40.6	79.2 ± 36.1	<0.001	13 (7–33)	56 (35–101)	0.138
AOC	16.3 (9.6–27.3)	39.5 (35.5–53.2)	—	65.9 ± 41.1	82.4 ± 37.4	—	13 (7–34)	61 (34–105)	—

Mean and interquartile range in parenthesis are shown for *ex vivo* experiments (non-normally distributed), mean and standard deviation are shown for healthy volunteers (normally distributed data), while median and range in parenthesis are shown for patients due to small sample size. P-value represents the comparison on SNR improvement between the different algorithms. Results are presented with AOC as a reference for comparison. For WSVD, SNR and SNR improvement were calculated after excluding data with negative SNR improvement from tumour specimens (N = 12) and healthy volunteers (N = 28). AOC = adaptively optimised combination. Nd-comb = noise decorrelation combination, WSVD = whitened singular value decomposition.

### AOC is independent from biochemical environment and voxel volume

**Figure 4:**
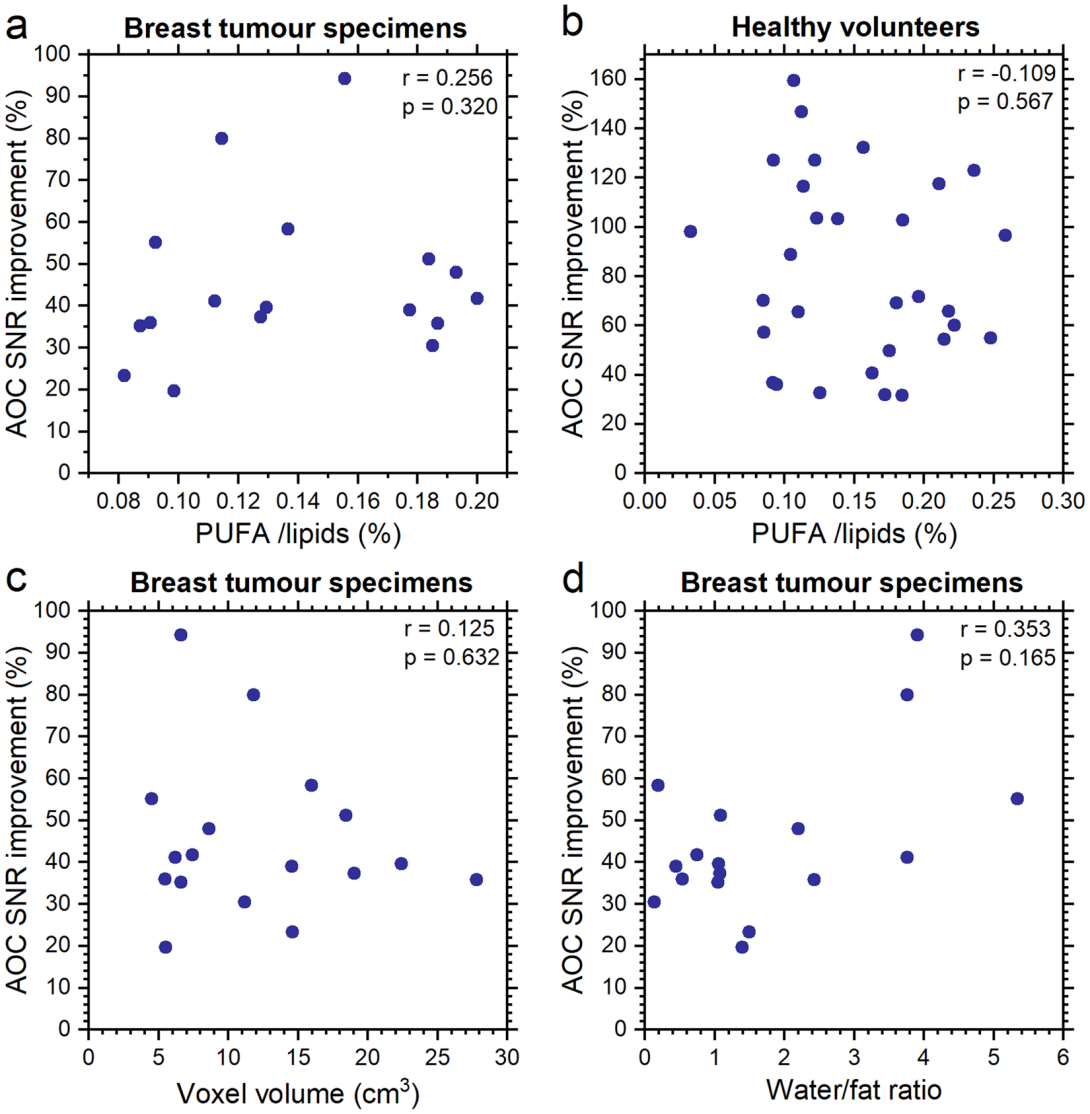
Spearman’s rank tests between SNR improvement and experimental parameters. The SNR improvement derived from AOC was correlated against (**a**) PUFA content for breast tumour specimens, (**b**) PUFA content for healthy volunteers, (**c**) voxel volume for breast tumour specimens, and (**d**) water/fat ratio for breast tumour specimens. Each dot represents an individual measurement. Spearman’s rho and p-value are displayed. Correlation against voxel volume for healthy volunteers was not conducted since the voxel remained constant across the entire cohort. Moreover, since the voxel was positioned in the adipose tissue, water signal was not detected. Therefore, correlation between SNR improvement and water/fat ratio was not conducted for healthy volunteers. AOC = adaptively optimised combination, PUFA = polyunsaturated fatty acids, SNR = signal to noise ratio.

We examined whether the SNR improvement, derived from AOC as being the optimal algorithm, is dependent on experimental conditions of PUFA content, voxel volume, and water/fat ratio. There was no significant correlation between the SNR improvement from AOC against PUFA content for breast tumour specimens (Fig. 4a) or healthy volunteers (Fig. 4b). The SNR improvement from AOC algorithm was independent from the voxel volume (Fig. 4c) and water/fat ratio in breast tumour specimens (Fig. 4d).

### Acquisition time in breast cancer using AOC

**Figure 5:**
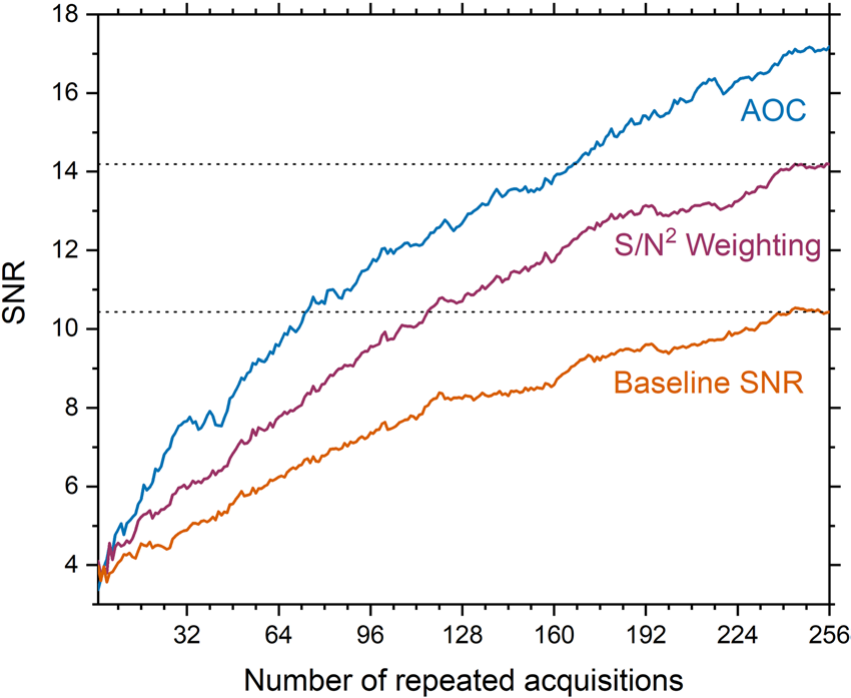
SNR as a function of the number of repeated acquisitions. The mean SNR obtained using AOC and S/N^2^ Weighting for PUFA spectra acquired from five patients is plotted as a function of the number of repeated acquisitions. Baseline SNR is also shown. The dotted line shows the maximum average baseline SNR (SNR = 10.4) and maximum average SNR using S/N^2^ Weighting (SNR = 14.1) after averaging for 256 acquisitions. To achieve the same SNR using AOC, the acquisition time can be reduced to 28% (6–43%) compared to baseline. If noise correlation is not ignored, the acquisition time can be reduced to 68% (46–72%) (1.5 times faster) compared to S/N^2^ Weighting. Values are given as median and range in parenthesis. Standard errors for each line are shown in Supplementary Fig. S2a,b.

We tested how much we can reduce the acquisition of PUFA spectra in patients if noise correlation is not ignored. Therefore, we examined how much we can reduce the scan time to achieve the same output SNR using AOC in comparison to S/N^2^ Weighting. We additionally compared AOC with Equal Weighting (baseline SNR). Our results show that using AOC, the number of repeated acquisitions for PUFA spectra (median, range) could be as low as 175 (117–185) or 71 (15–110) instead of 256 to achieve same output SNR as S/N^2^ Weighting and Equal Weighting respectively (Fig. 5, Supplementary Fig. S2a,b). Hence, PUFA acquisition time in patients could be reduced to 68% (46–72%) or to 28% (6–43%) of the original scan time compared to S/N^2^ Weighting and Equal Weighting respectively.

## Discussion

In this work, we compared current algorithms for combining PUFA spectra acquired using MQC MRS on phased array coils in breast cancer. AOC yielded the highest SNR improvement in excised breast tumours, healthy volunteers and patients. Moreover, AOC showed robustness against voxels with relatively low baseline SNR and was not affected by the presence of contamination lipid signal. AOC can be used for breast tumours with wide range of volumes, PUFA, water or fat contents, demonstrating the applicability in clinical settings.

AOC was the optimal algorithm consistently in both *ex vivo* and *in vivo* experiments. For *ex vivo* experiments, there was a clear difference in SNR improvement between the noise decorrelated algorithms (an average of 44% from AOC and nd-comb) and the linear algorithms (an average of 30% from S/N^2^ Weighting, S/N Weighting, and Signal Weighting). Among the linear algorithms, S/N^2^ Weighting had the best SNR improvement as predicted theoretically^9^ followed by S/N Weighting and Signal Weighting as shown in brain MRS^9^. WSVD gave a large variance of SNR improvement ranging from −100 to 77% dependent on the baseline SNR, since WSVD uses the PUFA spectra to estimate the weighting (reference spectra in AOC and nd-comb). A minimum baseline SNR (7.5 in this study) is required for WSVD to accurately identify the PUFA peak (phase and amplitude) in order to yield comparable SNR improvement as other noise decorrelated algorithms. This is in agreement with the literature findings in brain MRS where WSVD was shown to give comparable results with other noise decorrelated algorithms at high SNR levels but worse performance at low SNR^11^. AOC gave significantly higher SNR improvement compared to nd-comb, because nd-comb eliminates correlated noise before linear combination while AOC balances the signal against the overall noise^10^. For healthy volunteers, the difference in SNR improvement between the noise decorrelated algorithms and the linear algorithms was larger (up to 47%) due to higher noise correlation found *in vivo*. WSVD was susceptible to the presence of contamination lipid signal (for 4 out of 30 data), as found in brain MRS acquired near the skull^25^ whilst comparable to AOC for data with no lipid contamination. In patients, the difference between noise decorrelated algorithms and the linear algorithms was 28%. WSVD was susceptible to the presence of contamination signal in two patients and the low baseline SNR in the remaining three patients. AOC gave significantly higher SNR improvement compared to each of the algorithms apart from nd-comb. However, caution should be practised in interpreting these findings due to the small sample size. Based on consistent results among the experiments, AOC is most likely to provide the maximal SNR improvement in patients without susceptibility to low baseline SNR or contamination signal.

We examined the relationship between AOC and experimental conditions, including PUFA content, voxel volume and water/fat ratio. There was no dependency of AOC performance on voxel volume, and hence SNR level, in agreement with simulated and acquired MRS in the brain^11^. There was also no dependency of AOC on PUFA content and water/fat ratio in tumour specimens nor on PUFA content in healthy volunteers. In contrast, WSVD showed dependency on baseline SNR as well as voxel volume, confirming the categorical difference shown in group analysis. Therefore, AOC is functional in a wide range of breast tumour sizes and PUFA, water or fat contents, making it the ideal choice for clinical applications.

AOC, although independent from biochemical environment and voxel size in our study, is affected by voxel location^10^. Voxels, centred on the tumour, were positioned at the isocentre in *ex vivo* experiments. However, positioning the voxels at the isocentre was not feasible for *in vivo* experiments, leading to a wider signal disparity across coil elements, and in turn to a more pronounced SNR improvement, as shown in brain MRS studies^9,10^. The variability in SNR improvement observed *in vivo* was higher than *ex vivo* due to the varying voxel location across participants, and possibly the physiological differences across individuals. In patients, the variability in SNR and acquisition time improvement was additionally associated with the limited sample size of 5. Future studies will benefit from investigations on SNR and acquisition time improvement conducted on larger patient cohorts.

Enhanced SNR can be used to improve the accuracy of PUFA quantification, trade for reduced acquisition time, or enhance acquisition voxel volume. In comparison to Equal Weighting, AOC reduces acquisition time to approximately a quarter (median: 28%, range: 6–43% of original acquisition time) to maintain same output SNR, or improves SNR by 61% (34–105%) with same scan duration (equivalent to a reduction of voxel volume to 62% or 85% in each dimension). We also compared AOC with S/N^2^ Weighting as a non-noise decorrelated algorithm yielding the highest SNR in our study among the rest of the linear algorithms. In that case, acquisition is 1.5 times faster (AOC reduces acquisition time to 68% (56–71%) compared to S/N^2^ Weighting) to maintain same output SNR, or SNR is improved by 24% (4–28%) with same scan duration. These results show that noise correlation in breast MRS should be considered to achieve an optimised combination. A fast acquisition would be clinically valuable for elderly patients, who might experience difficulties remaining still in prone position or maintaining shallow breathing. Furthermore, the enhanced SNR would allow the assessment of small tumours more prevalent in breast cancer following the introduction of screening programmes. Reduced scan time would also be valuable for PUFA MR spectroscopic imaging (MRSI) when assessment of the whole diseased breast is necessary^4^. However, further analysis should be conducted to evaluate AOC performance on PUFA MRSI.

This study was limited on a single scanner and future work will benefit from multi centre studies on larger patient cohorts using scanners from different vendors, magnetic field strength, and breast coils. However, we deployed different coils for data collection between *ex vivo* and *in vivo* experiments with the results demonstrating the applicability of the algorithms under diverse conditions. The tumours studied in this work were limited to invasive ductal carcinoma with large size, and future investigation should be carried out in smaller tumours and other phenotypes. However, invasive ductal carcinoma accounts for approximately 75% breast cancer cases^26^, and the tumour size in this study is representative of typical patient population requiring MR scan.

AOC, among current algorithms, provided the maximum SNR improvement and is robust against low SNR or contamination signal. The derived SNR improvement was not dependent on experimental conditions of PUFA, water and fat content or voxel volume. Hence AOC is the optimal algorithm for PUFA spectra acquired using MQC MRS in breast to improve quantification accuracy, reduce acquisition time and reduce acquisition voxel volume.

## Methods

Experiments were conducted in freshly excised human breast tumours, healthy female volunteers and patients with breast cancer. The *ex vivo* and *in vivo* studies were approved by the North West – Greater Manchester East Research Ethics Committee (REC reference: 16/NW/0032) and the North of Scotland Research Ethics Service (REC reference: 16/NS/0077) respectively. All experiments were conducted in accordance with the Declaration of Helsinki guidelines and written informed consent was obtained from all participants prior to the study.

### *Ex Vivo* experiments

17 female patients (mean age, 61 years; age range, 42–78 years) with invasive ductal carcinoma (8 grade II and 9 grade III) and a tumour size greater than 10 mm in diameter were enrolled consecutively from Aberdeen Royal Infirmary hospital between February 2016 and June 2017. Patients having undergone chemotherapy or hormonal therapy were not eligible. The whole tumour, freshly excised with wide local excision or mastectomy, was placed in a container and immediately scanned, without formalin treatment, on a 3 T clinical MRI scanner (Achieva TX, Philips Healthcare, Best, Netherlands) using a body coil for uniform transmission and a 32-element phased array receiver coil for signal detection. Whole excised tissues were positioned in the centre of receiver coil. PUFA data were acquired using a single voxel MQC PRESS sequence^4^ with repetition time (TR) of 1250 ms, echo time (TE) of 130 ms, 1024 data points, spectral bandwidth of 2000 Hz, spectral editing frequency at 2.8 ppm and 256 averages. The scan time of PUFA acquisition was 5 minutes and 25 seconds. Reference spectra without water suppression were acquired using single voxel PRESS sequence^27^ with TR/TE of 1250/144 ms, 1024 data points, spectral bandwidth of 2000 Hz and 16 averages. The voxel was positioned to cover the whole tumour, with a voxel volume ranging from 2.7 to 16.5 cm^3^ according to tumour size. The noise in each coil element was obtained from an acquisition with no radiofrequency pulse applied.

### *In Vivo* experiments

15 healthy female volunteers (mean age, 66 years; age range, 58–76 years) with no history of breast cancer and 5 patients (mean age, 65 years; age range, 59–69 years) with invasive ductal carcinoma (3 grade II and 2 grade III) and a tumour size greater than 10 mm were recruited consecutively from North-East Scotland and Aberdeen Royal Infirmary respectively between September 2017 and August 2018. Only patients without chemotherapy or hormonal therapy were eligible. Spectra were acquired on a 3 T clinical MRI scanner (Achieva TX, Philips Healthcare, Best, Netherlands) using a body coil for uniform transmission and a 16-element phased array breast receiver coil for signal detection. The participants were in the prone position to minimise the effects of respiratory motion without respiratory gating, following standard clinical procedure. PUFA data were acquired using a single voxel MQC PRESS sequence^4^ with repetition time (TR) of 1250 ms, echo time (TE) of 130 ms, 1024 data points, spectral bandwidth of 2000 Hz, spectral editing frequency at 2.8 ppm and 256 or 128 averages for patients and volunteers respectively. Reference spectra without water suppression were acquired using single voxel PRESS sequence^27^ with TR/TE of 1250/144 ms, 1024 data points, spectral bandwidth of 2000 Hz and 16 averages. For volunteers, data were collected from both breasts with a voxel size of 2 × 2 × 2 cm^3^ containing mainly the adipose tissue. For patients, data were collected from the diseased breast with the voxel covering the tumour and voxel volume ranging from 2.6 to 8 cm^3^ according to tumour size. The noise in each coil element was obtained from an acquisition with no radiofrequency pulse applied.

### Data processing

Data were processed using in house software written in MATLAB (MathWorks, Natick, MA, USA), using fully automated literature methods (Table 1). The amplitude and phase shift were determined from the dominant peak (either water at 4.7 ppm or lipid at 1.3 ppm) in the reference spectrum. The SNR of each combined spectrum was defined as the PUFA peak height divided by the standard deviation of the real part of the spectrum within the range of 9.0–10.6 ppm^25^. The SNR improvement was defined as percentage increase of SNR referenced to the SNR derived from the Equal Weighting algorithm (signals are phased and equally weighted) as in line with literature^9^. The SNR derived from the Equal Weighting algorithm was defined as baseline SNR. Water (4.7 ppm), methyl fat (0.9 ppm) and methylene fat (1.3 ppm) from the reference spectrum and PUFA (5.3 ppm in MQC MRS) were quantified using AMARES algorithm^28^ in jMRUI software (v5.0, TRANSACT, Leuven, Belgium)^29^. PUFA content was expressed as percentage ratio of PUFA divided by the total lipids in reference spectrum (sum of methyl and methylene fat). Water to fat ratio was defined as the ratio of water divided by the total lipids. For each patient, 256 SNRs were further derived from raw data containing the corresponding number of repeated acquisitions using the optimal algorithm (AOC), the non-noise decorrelated algorithm with the highest improvement (S/N^2^ Weighting), and baseline SNR (Equal Weighting). As a result, the first SNR was derived from the first acquisition, the *n*th SNR from the first *n* spectra, and the 256th SNR from the entire 256 spectra. The mean SNR for each repeated acquisition was subsequently computed across all the 5 patients. As a result, the scan duration using the optimal algorithm was compared against non-noise decorrelation algorithms and baseline when same output SNR is maintained.

### Statistical analysis

Statistical analysis was performed in SPSS (Release 24.0, SPSS Inc., Chicago, USA). Shapiro-Wilk test was performed on the SNR improvement to assess normality. For normally distributed data, a repeated measures ANOVA test was performed to assess the group difference between the algorithms while Friedman and Wilcoxon signed-rank tests were used for non-normally distributed data. For patient data, with small sample size and an assumption of non-normal distribution (following indicatory pattern from breast tumour specimens), a Wilcoxon signed-rank test was performed. Data with negative SNR improvement were excluded for WSVD (5 out of 17 in breast tumour specimens and 2 out of 30 in healthy volunteers). Spearman’s rank tests were further performed to examine the dependency of the SNR improvement, derived from the optimal algorithm, on experimental conditions of PUFA content, voxel volume, and water/fat ratio. A p-value < 0.05 was considered as statistically significant. All statistical tests were two sided.

## Supplementary information

Supplementary Figures

## Data Availability

The datasets generated during and/or analysed during the current study are available from the corresponding author on reasonable request.
