# Supplementary material for: Optimal Phased-Array Signal Combination For Polyunsaturated Fatty Acids Measurement In Breast Cancer Using Multiple Quantum Coherence MR Spectroscopy At 3T
Source: Sci Rep. 2019 Jun 25;9:9259. doi: 10.1038/s41598-019-45710-1 (PMC6592938; doi:10.1038/s41598-019-45710-1)
Supplement: Supplementary file 1 — Supplementary Figures [file 41598_2019_45710_MOESM1_ESM.docx]

Title of manuscript: Optimal Phased-Array Signal Combination For Polyunsaturated Fatty Acids Measurement In Breast Cancer Using Multiple Quantum Coherence MR Spectroscopy At 3T

[First Author’s Surname:] Mallikourti

[Authors: name, highest degree, email]

1.*Vasiliki Mallikourti; MSc; r02vm16@abdn.ac.uk

1.Sai Man Cheung; PhD; g.cheung@abdn.ac.uk

2. Tanja Gagliardi ; PhD; tanja.gagliardi@abdn.ac.uk

3. Yazan Masannat; PhD; yazan.masannat@nhs.net

1,3. Steven D Heys; PhD; s.d.heys@abdn.ac.uk

1. Jiabao He; PhD; jiabao.he@abdn.ac.uk

**
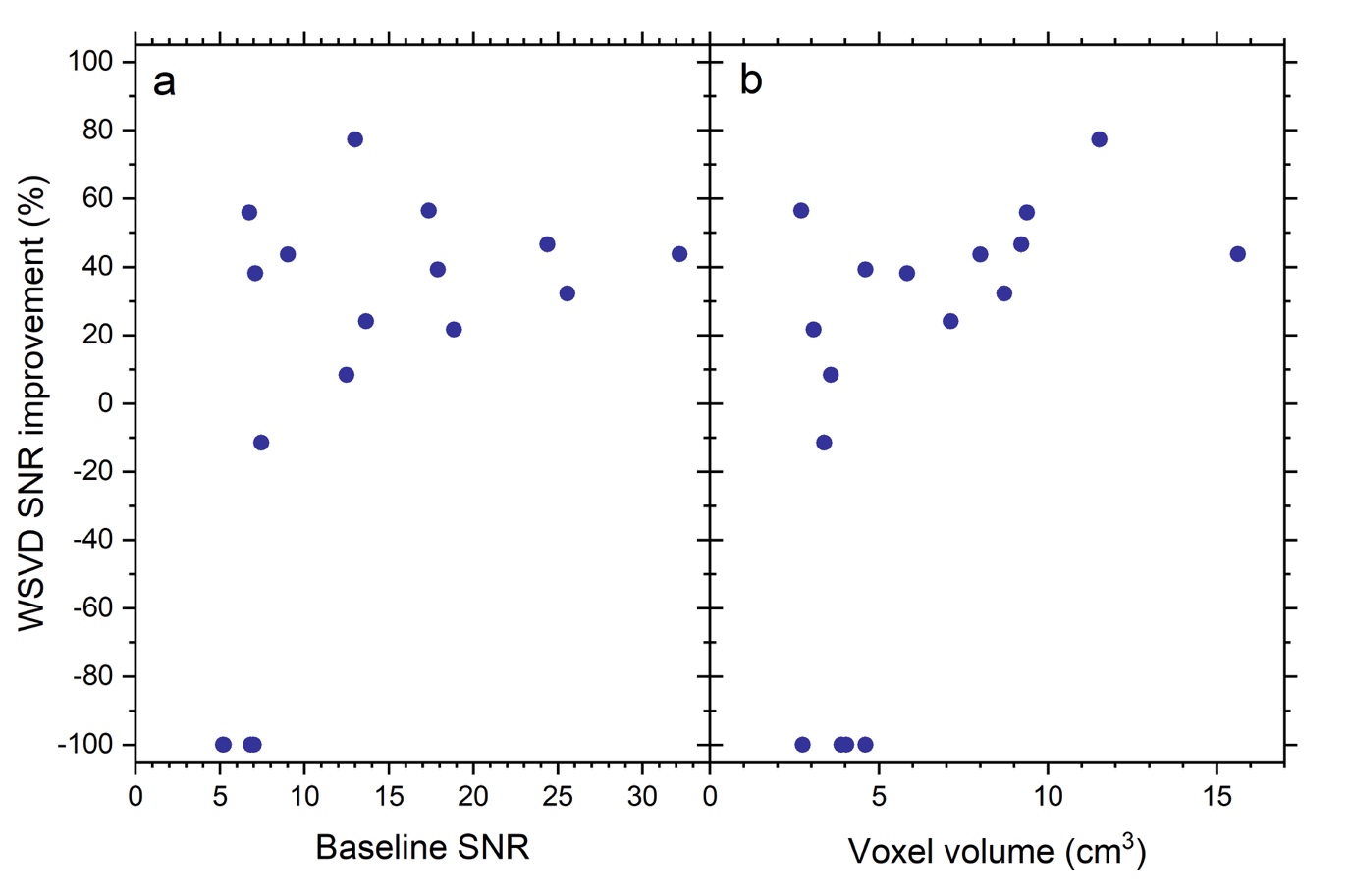
**

**Supplementary Figure S1. Dependency of WSVD on baseline SNR and voxel volume.**

SNR improvement from WSVD is plotted against (a) baseline SNR and (b) voxel volume in excised breast tumour specimens. Spearman’s rank test shows a significant correlation of WSVD against baseline SNR (rho = 0.515, p = 0.034) and voxel volume (rho = 0.556, p = 0.021). Each dot represents an individual measurement.


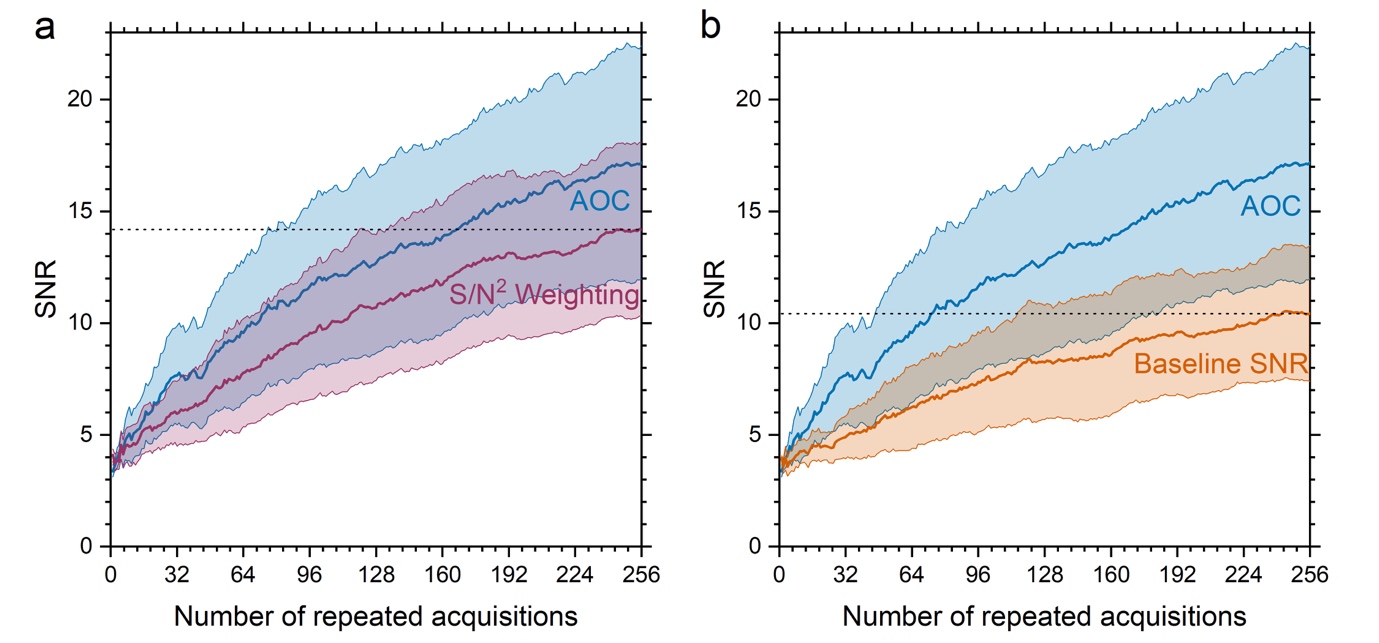

**Supplementary Figure S2. SNR as a function of the number of repeated acquisitions.**

SNR obtained using (a) AOC and S/N^2^ Weighting and (b) AOC and baseline SNR. Each line represents average SNR of PUFA spectra acquired from five patients while dash line shows the standard error at each number of repeated acquisitions.
